# The Carboxyl Terminus of Brca2 Links the Disassembly of Rad51 Complexes to Mitotic Entry

**DOI:** 10.1016/j.cub.2009.05.057

**Published:** 2009-07-14

**Authors:** Nabieh Ayoub, Eeson Rajendra, Xinyi Su, Anand D. Jeyasekharan, Robert Mahen, Ashok R. Venkitaraman

**Affiliations:** 1 Medical Research Council Cancer Cell Unit, Hutchison/MRC Research Centre, Hills Road, Cambridge CB2 2XZ, UK

**Keywords:** DNA

## Abstract

**Background:**

The Rad51 recombinase assembles on DNA to execute homologous DNA recombination (HR). This process is essential to repair replication-associated genomic lesions before cells enter mitosis, but how it is started and stopped during the cell cycle remains poorly understood. Rad51 assembly is regulated by the breast cancer suppressor Brca2, via its evolutionarily conserved BRC repeats, and a distinct carboxy (C)-terminal motif whose biological function is uncertain. Using “hit-and-run” gene targeting to insert single-codon substitutions into the avian *Brca2* locus, we report here a previously unrecognized role for the C-terminal motif.

**Results:**

We show that the avian C-terminal motif is functionally cognate with its human counterpart and identify point mutations that either abolish or enhance Rad51 binding. When these mutations are introduced into *Brca2*, we find that they affect neither the assembly of Rad51 into nuclear foci on damaged DNA nor DNA repair by HR. Instead, foci disassemble more rapidly in a point mutant that fails to bind Rad51, associated with faster mitotic entry. Conversely, the slower disassembly of foci in a point mutant that constitutively binds Rad51 correlates with delayed mitosis. Indeed, Rad51 foci do not persist in mitotic cells even after G2 checkpoint suppression, suggesting that their disassembly is a prerequisite for chromosome segregation.

**Conclusions:**

We conclude that Rad51 binding by the C-terminal Brca2 motif is dispensable for the execution of HR but instead links the disassembly of Rad51 complexes to mitotic entry. This mechanism may ensure that HR terminates before chromosome segregation. Our findings assign a biological function for the C-terminal Brca2 motif in a mechanism that coordinates DNA repair with the cell cycle.

## Introduction

Homologous DNA recombination (HR) plays an essential role in the resolution of replication-associated structures at replication forks and can act to repair single-stranded (ss) DNA gaps or double-stranded (ds) DNA breaks (DSBs) left in the wake of stalled or collapsed forks [1–4]. However, the DNA or protein structures that serve as intermediates in the DNA recombination process [5, 6], such as “X”-shaped DNA junctions or DNA-bound assemblies of recombination proteins, can pose an obstacle to mitosis. Mitotic entry in higher eukaryotes requires the physical concatenation and condensation of interphase DNA into compact chromosomes, processes likely to mandate the resolution or removal of recombination intermediates. Consistent with this notion, the artificial “tethering” of DNA damage-response proteins to chromatin has been reported to delay the cell cycle before mitotic entry [7].

The ordered nucleoprotein filament formed by multimerization of the eukaryotic DNA recombinase Rad51 on DNA substrates is essential for HR in vitro [8–10]. Visible microscopic Rad51 foci are formed within the cell nucleus following DNA damage [11], which contain large numbers of relatively immobile Rad51 molecules [12, 13] and may represent similar, DNA-bound Rad51 assemblies in vivo [14].

Rad51 multimerization is controlled by its interaction with the tumor suppressor Brca2 in vertebrates [15–17] or Brca2 orthologs in other complex eukaryotes [18]. In vertebrate species, Brca2 utilizes two motifs with apparently distinct properties to bind Rad51. An evolutionarily conserved region in the BRCA2 proteins encodes multiple BRC repeats [19], which bind directly to RAD51 [20] and possess apparently opposing activities in antagonizing RAD51 loading on dsDNA substrates while stabilizing RAD51-ssDNA interactions [21], which cooperate to promote DNA strand exchange [22, 23]. A second region at the extreme carboxyl (C) terminus of vertebrate Brca2, unrelated in its sequence to the BRC repeats, binds Rad51 [24, 25] in a manner regulated by the phosphorylation of a key serine residue (Ser3291 in human BRCA2) by cyclin-dependent kinases (CDKs) [26]. It has been proposed, primarily from biochemical studies, that the C-terminal RAD51-binding region of BRCA2 is essential for HR [26]. Ser3291 phosphorylation or its mutation to Ala or Glu leads to the dissociation of RAD51 from this binding site and the inability of a peptide spanning this region to protect RAD51 nucleoprotein filaments in vitro from antagonism by the BRC repeats [27, 28]. However, the in vivo biological function of the C-terminal RAD51-binding motif remains unclear.

Here, we have used “hit-and-run” gene targeting in avian DT40 cells, widely used for the study of DNA recombination [29–31], to introduce point mutations into an endogenous gene locus, *Brca2*. Our results show that single-codon substitutions that either abolish or enhance Rad51 binding to the C-terminal region of Brca2 do not measurably affect HR. Instead, our results reveal an unrecognized function of the C-terminal motif that links the dissolution of Rad51 complexes that serve as HR intermediates with entry into mitosis.

## Results

### The C-Terminal Motif of Avian *Brca2* Is Functionally Cognate with Its Human Counterpart in RAD51 Binding

**Figure 1:**
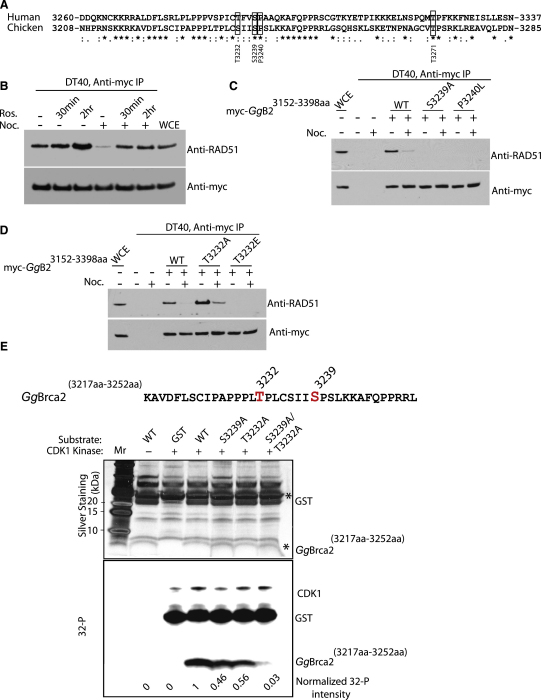
The C-Terminal Motif of *Gg*Brca2 Is Functionally Cognate with Its Human Counterpart in CDK-Regulated Rad51 Binding (A) Protein sequence alignment showing the conserved cyclin-dependent kinase (CDK)-phosphorylated residues flanking Ser3239 of *Gg*Brca*2* and Ser3291 of *Hs*BRCA2. *Gallus gallus* residues used in this study are boxed, and residue numbers are indicated below. Asterisks indicate identical residues, double dots indicate conserved substitutions, and single dots indicate residues that are semiconserved. (B) Western blot analysis of *Gg*Rad51 and the *myc* epitope following immunoprecipitation of the myc-tagged construct encoding residues 3152–3398 of *Gg*Brca2 (*Gg*B2^3152 aa–3398 aa^) transfected into DT40 cells. Roscovitine treatment for 30–120 min in asynchronous cells had little effect on *Gg*Rad51 binding (lanes 2 and 3). However, roscovitine effectively reversed the reduction in binding induced by nocodazole (compare lanes 5 and 6 with lane 4). (C and D) Western blot analysis of *Gg*Rad51 and the *myc* epitope following immunoprecipitation comparing the wild-type (WT) with S3239A/E or P3240L (C) or T3232A (D) variants of *Gg*B2^3152 aa–3398 aa^ transfected into DT40 cells. The S3239A/E and P3240L mutations in the conserved Ser-Pro consensus for CDK phosphorylation abrogated binding to *Gg*Rad51 in asynchronous and nocodazole-arrested mitotic cell extracts, whereas the T3232A mutation caused enhanced binding to *Gg*Rad51 under the same conditions. (E) Thr3232 and Ser3239 can be phosphorylated in vitro by CDK1. A wild-type *Gg*Brca2 peptide fused with GST (WT, sequence at top) or mutant forms in which Thr3232 (T3232A), Ser3239 (S3239A), or both (T3232A/S3239A) were substituted with Ala were subjected to an in vitro kinase assay in the presence of [γ-^32^P]ATP. Reaction products were cleaved with thrombin to separate the Brca2 peptides from GST. Silver staining (middle panel) measures the loading of the peptides in each reaction (^∗^). The bottom panel shows that CDK1 catalyzes the transfer of ^32^P radiolabel from [γ-^32^P]ATP to the T3232A or S3239A peptides, but not to the double-mutant T3232A/S3239A peptide. Numbers at bottom indicate relative phosphorylation normalized to the amount of peptide present in each sample.

Three lines of evidence indicate that the C-terminal Rad51-binding motif in *Gallus gallus* (*Gg*) *Brca2* is functionally cognate with the corresponding region in *Homo sapiens* (*Hs*) *BRCA2*. First, sequence alignment with ClustalW [32] followed by EMBOSS pairwise-alignment algorithms [33] shows considerable evolutionary conservation (Figure 1A). The candidate CDK phosphorylation sites Thr3232, Ser3239, and Thr3271 of *GgBrca2* correspond to Ser3284, Ser3291, and Thr3323 of *HsBRCA2* (Figure 1A). They are embedded in a 78-residue sequence spanning avian residues 3208–3285 (corresponding to human residues 3260–3337). This motif in *GgBrca2* is 42.3% identical and 66.7% similar to its human counterpart (compared to 34.3% identity and 49.9% similarity over the entire avian and human Brca2 molecules). Significantly, the relative position of other key residues is also preserved. Second, the C-terminal motif of *Gg*Brca2, like the cognate region from *Hs*BRCA2 [26], binds to Rad51 in interphase cell extracts, but not in extracts from nocodazole-arrested cells (Figure 1B, compare lane 4 with lanes 1–3). Third, as in the corresponding human region [26], the loss of *Gg*Rad51 binding in nocodazole-arrested avian cells is promoted by CDK phosphorylation. Treatment with the CDK inhibitor roscovitine for 30–120 min [34] prevents the nocodazole-induced release of *Gg*Rad51 from the C-terminal region of avian Brca2 [26] (Figure 1B).

### Mutational Analysis of the C-Terminal Rad51-Binding Motif of Avian *Brca2*

Further underscoring their functional conservation, we found that the both human and avian Brca2 share a conserved Ser-Pro consensus for CDK phosphorylation in their C-terminal motifs. A *myc* epitope-tagged form of the wild-type C-terminal fragment of *Gg*Brca2 or point mutants in which the Ser or Pro residues were substituted were immunoprecipitated from extracts of transfected avian DT40 cells to examine their ability to bind Rad51. Similar to *Hs*BRCA2 [26], changing Ser3239 to Ala or Glu or substituting Pro3240 with Leu led to the failure of *Gg*Rad51 binding (Figure 1C). The effect of these point mutations on Rad51 binding was not altered by nocodazole-induced mitotic arrest (Figure 1C).

Additional residues in the C-terminal region of *Hs*BRCA2 also undergo CDK-dependent phosphorylation [26]. Accordingly, we examined the effect on Rad51 binding of mutations affecting other putative phosphorylation sites in *Gg*Brca2 (Figure 1D). Interestingly, mutation of Thr3232 to Ala markedly enhanced the binding of *Gg*Rad51 to the C terminus of *Gg*Brca2 even after nocodazole treatment. On the other hand, the alteration of Thr3232 to Glu abolished binding of *Gg*Rad51, suggesting a critical role for phosphorylation of this residue in regulating the interaction between *Gg*Rad51 and the C-terminal *Gg*Brca2 motif.

The sequences surrounding residues Thr3232 and Ser3239 in *Gg*Brca2, like their counterparts in *Hs*BRCA2 (Figure 1A), only weakly match the consensus substrate motif for CDKs. We therefore used ^32^P-labeled γ-ATP to determine whether they could be phosphorylated by recombinant CDK1 in vitro. A GST-tagged *Gg*Brca2 fragment spanning residues 3217–3252 and mutant forms carrying T3232A, S3239A, or T3232A/S3239A substitutions were tested. Our results confirmed that CDK1 can phosphorylate Thr3232 and Ser3239 in vitro (Figure 1E).

Collectively, the results shown in Figure 1 provide firm evidence that the C-terminal Rad51-binding motif in *Gg*Brca2 is functionally cognate with the corresponding region in its human counterpart and identify Thr3232 as a new determinant of Rad51 binding.

### Targeted Replacement of Ser3239, Pro3240, and Thr3232 in Avian *Brca2*

Next, we used somatic gene targeting [35] to individually substitute the Ser3239, Pro3240, and Thr3232 residues in avian DT40 cells. For the homozygous knockin of the S3239A point mutation, targeting constructs with a diagnostic restriction site (SacI) and a *lox*P-flanked blasticidin or neomycin resistance gene (see Figure S1A available online) were sequentially transfected into wild-type DT40 cells. The S3239A substitution was confirmed by polymerase chain reaction (PCR) and restriction digestion (Figure S1B).

We then devised a hit-and-run approach using *Cre*-mediated recombination [36] to remove extraneous sequences and restore the structure of the *GgBrca2* gene. The S3239A homozygous cell line (*Brca2^S3239A/S3239A^*) was transfected with a plasmid encoding Cre recombinase. In cells with restored sensitivity to Bsr and Neo, S3239A knockin was further verified by sequencing the corresponding DNA region amplified by reverse transcriptase-PCR (data not shown). Western blots for *Gg*Brca2 in the wild-type (WT) and Gg*Brca2^S3239A/S3239A^* mutant cell lines showed comparable levels of the protein, confirming that S3239A mutation has no significant effect on protein expression or stability (Figure S1C).

Alteration of Pro3292 to Leu in human *BRCA2* has been reported to occur in familial breast cancer cases (Breast Cancer Information Core, BIC database; http://research.nhgri.nih.gov/bic/). Given that the second *BRCA2* allele is consistently deleted from cancer cells [37, 38], we used gene targeting to introduce the corresponding P3240L mutation into one allele of *GgBrca2*, while deleting the second allele, to recapitulate cancer-associated genetic alterations (Figures S2A and S2C). A *GgBrca2* heterozygous cell line was created by using two targeting constructs with *Bsr* and *Neo* resistance genes to sequentially knock in two *lox*P sites flanking the entire genomic coding sequence of *Brca2*, one upstream of the initiating ATG codon and the second downstream of the stop codon (Figure S2A). Pulsed-field gel electrophoresis and Southern blotting were used to validate successful targeting at both sites. As before, a hit-and-run approach was then used to delete the 40 kb region between the *lox*P sites (the “floxed” region) containing one entire *GgBrca2* allele along with the drug-resistance cassettes used for gene targeting (Figure S2B). Substitution of Pro3240 with Leu or Thr3232 with Ala in the heterozygous *GgBrca2* cell line employed the same strategy used for engineering the S3239A mutation (Figure S1A). Successful knockin of the P3240L and T3232A substitutions was confirmed by PCR and restriction digestion (Figures S2C and S2D).

Western blotting revealed a comparable level of Brca2 protein in the *Brca2^+/−^*, *Brca2^P3240L/−^*, and *Brca2^T3232A/−^* cell lines, approximately half the amount in wild-type cells (Figure S2E). These results provide a first example of the introduction of point mutations by hit-and-run targeting of endogenous gene loci in DT40 cells

### Rad51 Binding to the C-Terminal Brca2 Motif Is Dispensable for Gene Conversion by HR

**Figure 2:**
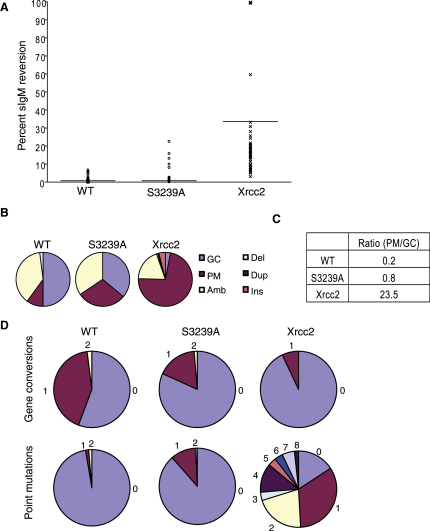
Immunoglobulin Gene Diversification in the Mutant Cell Line *Brca2^S3239A/S3239A^* Confirms the Integrity of Homologous DNA Recombination (A) Luria-Delbrück fluctuation analysis shows no significant difference in sIgM+ reversion to sIgM− status between the WT and S3239A cell lines. Xrcc2-deficient cells show a significant increase in median fluctuation relative to WT and S3239A (median percentage reversion indicated by horizontal line: WT 0.67%, S3239A 0.57%, Xrcc2 17.6%). (B) Pie charts showing that the source of mutation in VL1 is predominantly gene conversion in WT and *Brca2^S3239A/S3239A^* cells but predominantly point mutation in Xrcc2-deficient cells (n = 149, 148, and 54, respectively). (C) Table showing the ratio of point mutation to gene conversion suggests that commitment to homologous DNA recombination (HR) (gene conversion) is intact in *Brca2^S3239A/S3239A^* cells but has shifted to nontemplated error-prone repair pathways (point mutation) in Xrcc2-deficient cells. (D) Pie charts showing frequencies of unique gene conversion and point mutation events in WT and *Brca2^S3239A/S3239A^* cells indicate a preference for HR over the point mutation used extensively in Xrcc2-deficient cells.

It has been suggested from biochemical data that RAD51 binding to the C-terminal region of BRCA2 is necessary for HR [26]. To directly test this idea, we asked whether a mutation that prevents *Gg*Rad51 binding to the C terminus of *Gg*Brca2 (*Brca2^S3239A/S3239A^*) affects the integrity of gene conversion events in vivo (Figure 2). We monitored mutation in the immunoglobulin gene locus, which constitutively undergoes activation-induced deaminase-dependent hypermutation in the absence of exogenous DNA-damaging agents [31]. The immunoglobulin gene diversification assay is used to determine the rate and mechanism of mutations that render DT40 cells unable to express surface immunoglobulin (sIg), thus reporting events in a specific, endogenous gene locus [39]. In wild-type cells, generation of sIgM− clones from an initially sIgM+ population occurs predominantly through templated mutation from pseudo-V genes (gene conversion, which requires HR). Less frequently, nontemplated mutation (point mutation) may also generate sIgM− clones. DT40 mutants deficient in HR exhibit a shift from templated gene conversion to nontemplated point mutations [29].

To test the integrity of HR in *Brca2^S3239A/S3239A^* cells, a Luria-Delbrück fluctuation analysis of the conversion of sIgM+ cells to sIgM− (to determine the rate of mutation) was combined with sequencing and analysis of the VL1 locus in sIgM− cells (to determine the mechanism of mutation). The Luria-Delbrück analysis showed no significant difference between wild-type and *Brca2^S3239A/S3239A^* cell lines (p > 0.05, two-tailed unpaired t test), implying that the S3239A does not change the rate of mutation. By contrast, an Xrcc2-deficient cell line, known to be defective in HR, exhibited a significant increase in fluctuation relative to WT and *Brca2^S3239A/S3239A^* cells (Figure 2A) [39].

The VL1 locus in sIgM− cells was then sequenced to determine the mechanism of mutation. The proportions of each type of mutation (i.e., gene conversion versus point mutation versus ambiguous changes) in VL1 were similar in WT and *Brca2^S3239A/S3239A^* cells. Again, this contrasted with the marked shift to point mutation in cells lacking Xrcc2 (Figures 2B and 2C). A more detailed analysis of the profile of mutations within each sequence in each cell line confirmed that like WT cells, *Brca2^S3239A/S3239A^* cells exhibit a low rate of mutation. This was indicated not only by the low fluctuation rate but also by the high proportion of unmutated sequences. In comparison, Xrcc2-deficient cells showed a high rate of fluctuation; conversion to the sIgM− state occurred predominantly via point mutation instead of gene conversion, and the proportion of VL1 sequences containing more than one point mutation was also high (Figure 2D).

Collectively, our results provide strong evidence that abrogation of the interaction between Rad51 and the C-terminal region of *Gg*Brca2 does not detectably impair HR. By contrast, it has previously been shown that truncation of *Gg*Brca2 within the region encoding the BRC repeats phenocopies the defective HR seen in Xrcc2-deficient cells [29]. We therefore infer that whereas the interaction of Rad51 with the BRC repeats is essential for the early events in HR, most notably Rad51 loading and promotion of the strand exchange reaction, the interaction of Rad51 with the C-terminal region of Brca2 is not.

### HR Is Unimpaired by Mutations that Prevent or Enhance Rad51 Binding to the C-Terminal Motif of Brca2

Next, we asked whether mutations that prevent (*Brca2^S3239A/S3239A^* and *Brca2^P3240L/−^*) or enhance (*Brca2^T3232A/−^*) *Gg*Rad51 binding to the C terminus of *Gg*Brca2 affect cellular sensitivity to exogenous DNA damage inflicted by ionizing radiation (IR), the DNA crosslinking agent mitomycin C (MMC), or the topoisomerase I inhibitor camptothecin, repair of all of which requires HR. However, we observed no significant impairment in the ability of *Brca2^S3239A/S3239A^*, *Brca2^P3240L/−^*, and *Brca2^T3232A/−^* cells to survive exposure to any of these agents in comparison to wild-type controls (Figure S3), suggesting that HR was intact.

**Figure 3:**
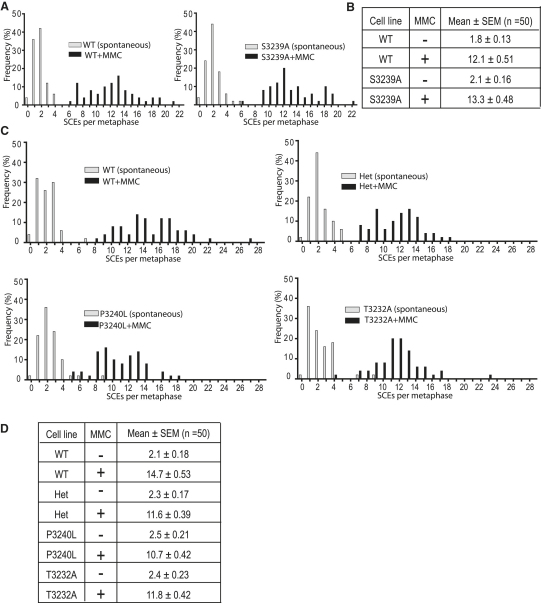
No Defect in Spontaneous or Mitomycin C-Induced Sister Chromatid Exchanges in the *Brca2^S3239A/S3239A^*, *Brca2^+/−^*, *Brca2^P3240L/−^*, and *Brca2^T3232A/−^* Cell Lines (A) Frequency distribution histograms of spontaneous (gray) and mitomycin C (MMC)-induced (black) sister chromatid exchanges (SCEs) in WT and *Brca2^S3239A/S3239A^* cell lines show significant induction in both (two-tailed unpaired t test, n = 50). (B) The mean ± SEM of each frequency distribution in (A) is indicated. Contrary to prediction, no significant diminution of basal or MMC-induced SCEs in *Brca2^S3239A/S3239A^* cells was observed. Fold induction from SCE levels in unchallenged cells is 6.7 in WT compared to 6.3 in S3239A mutant. (C) Frequency distribution histograms of spontaneous (gray) and MMC-induced (black) SCEs in WT, *Brca2^+/−^*, *Brca2^P3240L/−^*, and *Brca2^T3232A/−^* cell lines show a significant induction of SCE by MMC in all cell lines (two-tailed unpaired t test, n = 50). Both *Brca2^T3232A/−^* and *Brca2^P3240L/−^* cells exhibit basal and MMC-induced SCE comparable to their parental heterozygous *Brca2^+/−^* cells. (D) Table enumerating SCEs (mean ± SEM) in unchallenged or MMC-treated cells. Heterozygous cells and mutants derived from them have basal levels of SCEs similar to WT cells, but modestly less induction after MMC treatment. Fold inductions are: WT, 7.0; Het, 5.1; P3240L, 4.4; T3232A, 5.0.

The proper initiation and prosecution of HR is also required for the formation of sister chromatid exchanges (SCEs) during the mitotic cell cycle and their elevation after challenge with MMC. The basal and MMC-induced measurement of SCEs is therefore widely used as a sensitive and specific test for the integrity of homologous DNA recombination pathways [40]. Consistent with the lack of genotoxin sensitivity, we did not observe any significant effect on basal or MMC-induced SCEs of mutations that affect Rad51 binding to the C-terminal motif of Brca2 (Figures 3A–3D). The frequency distribution of basal SCE number in metaphases from unchallenged wild-type, *Brca2^S3239A/S3239A^*, *Brca2^+/−^*, *Brca2^P3240L/−^*, or *Brca2^T3232A/−^* cells was virtually overlapping, and there was no significant difference in the mean values (p > 0.05 by two-way ANOVA with Bonferroni post hoc test) (Figures 3A–3D).

Collectively, our findings provide multiple lines of evidence suggesting that the interaction of Rad51 with the C-terminal motif of Brca2 is dispensable for the execution of HR.

### Rad51 Binding by the C-Terminal Brca2 Motif Affects the Disassembly of Damage-Induced Rad51 Foci

Recent biochemical work [15, 28] suggests that RAD51 binding to the C-terminal region of BRCA2 affects the stability of DNA-bound RAD51 filaments in vitro. In vivo, such structures may be represented by microscopically visible RAD51 foci, which have been shown to assemble at the sites of induced DSBs and colocalize there with resected ssDNA marked by BrdU incorporation [41–43]. We therefore examined Rad51 focus formation in cell lines harboring point mutations in the C-terminal Rad51-binding motif of avian Brca2. Mutant cells with either reduced (*Brca2^P3240L/−^*) or enhanced (*Brca2^T3232A/−^*) binding of Rad51 to the C terminus of Brca2 displayed a similar induction in the average number of Rad51 foci per cell 3 hr after IR-induced damage compared to the control parental cell line (Figure S4; Figure S5). This suggests that the interaction of Rad51 with the C-terminal motif of Brca2 is dispensable for the reactions that lead to Rad51 assembly at sites of DNA damage and is consistent with our observation that HR is not detectably impaired (Figure 2; Figure 3).

However, there was a statistically significant increase in the mean number of Rad51 foci per cell in the *Brca2^T3232A/−^* cell line, even in the absence of DNA damage (Figure S5). Given that this mutant cell line, like the others tested here, exhibited no alteration in its ability to form damage-induced Rad51 foci, this raised the possibility that the T3232A mutation might promote the persistence of Rad51 foci after their formation. This reasoning prompted us to examine the kinetics of the disappearance of Rad51 foci in the mutant cell lines after exposure to DNA damage.

**Figure 4:**
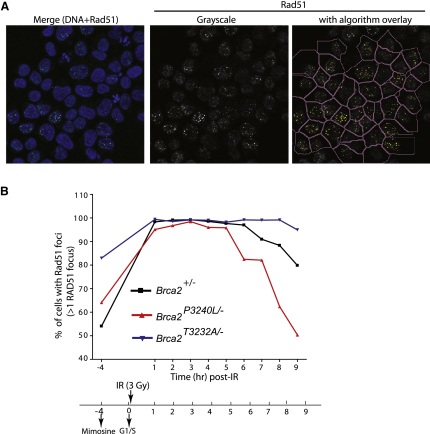
Normal Formation but Altered Dissolution of Damage-Induced Rad51 Foci in *Brca2^P3240L/−^* and *Brca2^T3232A/−^* Cells (A) Representation of a spot-counting algorithm on the Cellomics ArrayScan VTI. Left panel: a merged image of DNA (blue) and Rad51 (green). Middle and right panels: grayscale image of Rad51 exported to the Cellomics ArrayScan platform with (middle) and without (right) the algorithm overlay (details in Experimental Procedures). (B) Kinetics of Rad51 focus formation and dissolution in mimosine-synchronized *Brca2^+/−^*, *Brca2^P3240L/−^*, and *Brca2^T3232A/−^* cells after exposure to 3 Gy ionizing radiation (IR). Five hundred cells were analyzed on the Cellomics ArrayScan for each data point, and the percentage of cells positive for Rad51 foci was plotted as a function of time (see Experimental Procedures for details). Nearly 100% of cells formed Rad51 foci after IR in all three cell lines by 3 hr after damage. However, decreased binding to Rad51 in the *Brca2^P3240L/−^* cell line correlated with a faster reduction in focus-positive cells compared to the heterozygous control. Conversely, the *Brca2^T3232A/−^* cell line showed a slower reduction in foci dissolution.

*Brca2*^+/−^, *Brca2^P3240L/−^*, and *Brca2^T3232A/−^* cells synchronized in G1 and released into S phase were exposed to 3 Gy IR immediately after release and harvested every hour afterwards for analysis. Rad51 focus formation was enumerated in 500 cells for each data point by automated high-content microscopy (Figure 4A). The percentage of cells containing Rad51 foci was plotted as a function of time (Figure 4B). In all cell lines, nearly 100% of cells contained Rad51 foci by 3 hr after IR. However, in the *Brca2^P3240L/−^* cell line, there was a faster reduction in the percentage of focus-positive cells relative to wild-type, evident 4–5 hr after IR exposure. The P3240L mutation prevents Rad51 binding to the C-terminal Brca2 motif, suggesting that the loss of the interaction promotes this change. Consistent with this, accelerated dissipation of IR-induced Rad51 foci was also observed in *Brca2^S3239A/S3239A^* cells (Figure S6). This notion was further supported by the converse effect of the T3232A mutation, which enhanced Rad51 binding. In *Brca2^T3232A/−^* cells, there was a slower reduction in the percentage of focus-positive cells (Figure 4B). Together, these observations suggest that the C-terminal Rad51-binding motif in Brca2 regulates not the assembly of Rad51 foci at sites of DNA damage but rather the rate of their dissolution.

### Absence of Rad51 Foci in Mitotic Cells

We reasoned that because HR occurs during the late S or G2 phases of the cell cycle [44, 45], the removal of Rad51 assemblies from DNA might facilitate the structural changes to chromosomes that accompany mitosis [46, 47]. If correct, this would predict that visible Rad51 foci, markers of its assembly at the sites of HR, should be absent from mitotic cells.

**Figure 5:**
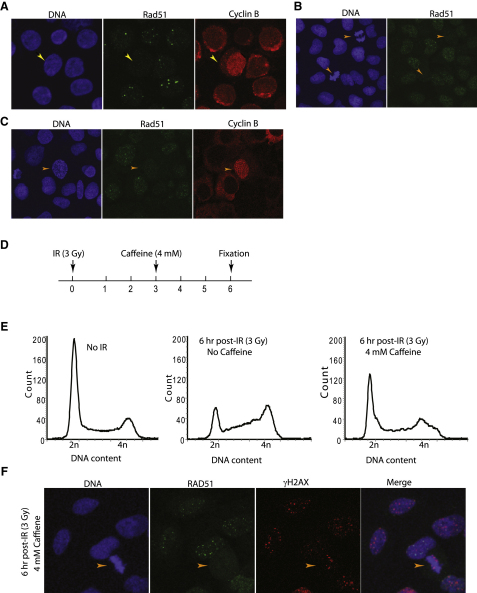
Rad51 Foci Are Absent from Mitotic Cells (A) Immunofluorescence analysis of asynchronous WT DT40 cells costained for Rad51 (green) and cyclin B (red). A typical prophase cell (yellow arrowhead) shows intense nuclear (but not cytosolic) staining of cyclin B and is negative for Rad51 foci. DNA is stained with DAPI (blue). (B and C) Immunofluorescence staining of asynchronous human U2OS cells for RAD51 (green) and/or cyclin B (red). RAD51 foci are absent from U2OS cells marked by condensed DAPI-stained chromosomes that have entered mitosis (orange arrowheads). (D) Schematic of the experimental timeline. (E) Cell-cycle profiles of asynchronous, irradiated, and irradiated plus caffeine-treated U2OS cells. DNA content measured by propidium iodide staining and flow cytometry is plotted on the horizontal axis against relative cell number. 2n and 4n peaks represent the G1 and G2/M phases, respectively. (F) Representative immunofluorescence micrographs confirming the absence of RAD51 foci (green) in a mitotic cell (arrowhead) in which γH2AX staining (red) persists after exposure to IR and caffeine. DNA was stained with DAPI (blue). No Rad51 foci were observed in any of 50 mitotic nuclei identified by DAPI staining, whereas γH2AX staining persisted in 38 (76%) of them.

We present several lines of evidence that this is indeed the case (Figure S7; Figures 5A–5C). First, in a population of DT40 cells wild-type for Rad51 passing synchronously through the cell cycle (Figure S7A), there was an inverse correlation as they entered mitosis between staining for the mitotic marker anti-MPM2 and the presence of Rad51 foci, suggesting that these markers are mutually exclusive (Figure S7B). Second, we found that individual cells that have entered mitosis rarely contain Rad51 foci. To test this, we costained cells for Rad51 and cyclin B, which becomes nuclear at the onset of nuclear envelope breakdown during prophase [48]. Strikingly, 98% of prophase DT40 cells (n = 100), marked by nuclear cyclin B localization, were negative for Rad51 foci (Figure 5A). The absence of Rad51 foci during mitosis was also a feature in a human cell line. Human U2OS cells in the mitotic prometaphase, metaphase, or anaphase, marked by a typical pattern of DAPI staining, were devoid of RAD51 foci (Figure 5B). As in wild-type DT40 cells, entry into prophase marked by nuclear cyclin B localization was consistently accompanied by the absence of RAD51 foci (Figure 5C). Collectively, these findings suggest that entry into mitosis in both avian and human cells is accompanied by the dissolution of nuclear foci containing RAD51.

Interestingly, the absence of RAD51 foci from mitotic cells was evident even after their exposure to IR (Figures 5D–5F). Asynchronously dividing U2OS cells exposed to 3 Gy IR were treated 3 hr afterwards with 4 mM caffeine (Figure 5D). The cell-cycle profile marking asynchronous division (Figure 5E, left panel) was altered after IR exposure to accumulation with 4n DNA content in the G2 phase (Figure 5E, middle panel), with only 0.1% of cells entering mitosis as evinced by nuclear staining for cyclin B (data not shown). Caffeine overcame the G2 checkpoint (Figure 5E, right panel), allowing premature release into mitosis, leading to a 10-fold increase in the number of cells in which cyclin B staining was nuclear. Strikingly, we observed that IR-exposed, caffeine-treated U2OS cells were consistently devoid of RAD51 foci during mitosis, even when DNA damage marked by γH2AX staining still persisted (Figure 5F). After caffeine-mediated release into mitosis following IR exposure, immunofluorescence analysis revealed no mitotic nuclei containing RAD51 foci, whereas γH2AX phosphorylation persisted in 76% of the cells (n = 50). These findings suggest not only that the clearance of RAD51 foci is a prerequisite for mitotic entry, even in cells exposed to DNA damage, but also that the mechanism is independent of caffeine-sensitive G2 checkpoint kinases.

### Mutations that Alter Rad51 Binding by the C-Terminal Brca2 Motif Regulate Mitotic Entry Coordinately with Rad51 Foci Dissolution

We have shown that C-terminal Brca2 mutations preventing Rad51 binding accelerate the dissolution of damage-induced Rad51 foci, and vice versa (Figure 4B; Figure S6). Given the inverse relationship between mitotic entry and Rad51 focus clearance (Figure 5; Figure S7), we reasoned that the C-terminal motif in Brca2 might regulate their coordination.

**Figure 6:**
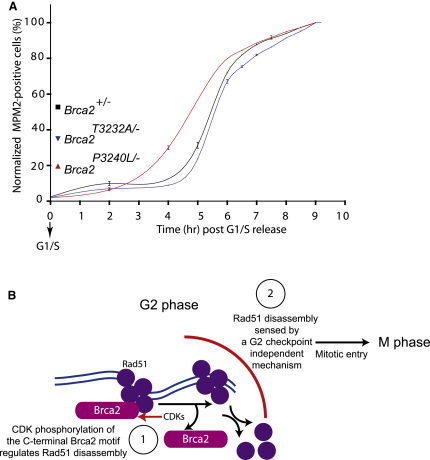
Coordinate Regulation of Mitotic Entry with Rad51 Binding by the C-Terminal Brca2 Motif (A) *Brca2^+/−^*, *Brca2^P3240L/−^*, and *Brca2^T3232A/−^* cells were synchronized with mimosine at the G1/S boundary and released into S phase. Samples collected at the indicated time points were stained with anti-MPM2 and analyzed by flow cytometry. The percentage of MPM2-positive cells is plotted on the vertical axis against time after release from G1/S. Each data point represents a mean ± SEM percentage from three samples. MPM2 staining marks cells entering mitosis, which usually increases 4 hr after release from G1/S in control *Brca2*^+/−^ parental cells (Figure S7B). In the *Brca2^P3240L/−^* cell line, this increase is hastened, whereas conversely, in the *Brca2^T3232A/−^* cell line, it is delayed. (B) A hypothetical model for the biological role of the C-terminal Brca2 motif in coordinating DNA repair by HR with mitotic entry. During the G2 phase, phosphorylation of the C-terminal Brca2 motif by CDKs regulates the disassembly of DNA-bound Rad51 complexes (1), a process monitored by a mechanism that is independent of the G2 checkpoint for DNA damage (2). Completed disassembly allows entry into the M phase.

To test this notion, *Brca2^+/−^*, *Brca2^P3240L/−^*, and *Brca2^T3232A/−^* cells synchronously passing through S phase were analyzed hourly for DNA content by propidium iodide staining and concurrently with an anti-MPM2 antibody as a marker for entry into mitosis. Strikingly, in the *Brca2^P3240L/−^* mutant, the increase in MPM2-positive cells began 2 hr after release into S phase, rather than at 4–5 hr as in *Brca2^+/−^* parental cells, suggesting early entry into mitosis (Figure 6A). Conversely, the index of MPM2 staining was delayed in the *Brca2^T3232A/−^* mutant cell line compared to the control. In both cases, Rad51 foci were not present in mitotic cells (data not shown). The altered timing of mitotic entry in the Brca2 mutant cells was statistically significant in pairwise comparisons with the control (p < 0.01, two-way ANOVA). Together, these experiments suggest that Rad51 binding to the C-terminal motif of Brca2 coordinates mitotic entry with the dissolution of Rad51 foci.

## Discussion

Our findings address the in vivo biological function of the C-terminal Rad51-binding motif in Brca2, using a system of hit-and-run gene targeting to introduce point mutations into the avian *Brca2* locus that alter CDK-phosphorylated residues cognate with counterparts in human BRCA2, affecting the RAD51 interaction in vitro. We show that neither mutations that prevent nor a mutation that enhances Rad51 binding in vitro have a measurable effect on HR in vivo. From this, we infer that the interaction between Rad51 and the C-terminal motif in Brca2 is dispensable for homologous DNA recombination, at least in this vertebrate model for mitotic recombination. Our conclusions are consistent with the recent observation that a “minimal” BRCA2 protein comprising one or more BRC repeats fused to the DNA-binding domain of replication protein A (RPA) suffices to complement the IR sensitivity of *BRCA2*-deficient cells [49, 50] and the fact that point mutations in the C terminus of human BRCA2, including S3291A and P3292L, fail to show a discernable defect in DNA repair [51]. Together, these findings highlight a seeming mechanistic distinction between the two types of Rad51-binding motifs in vertebrate Brca2: the BRC repeats, which are apparently essential for HR, versus the C-terminal motif, which is not.

Instead, our work indicates that the interaction between Rad51 and the C-terminal motif of Brca2 serves an alternative purpose (Figure 6B). We show that mutations that prevent Rad51 binding to the C-terminal motif in Brca2 lead to the faster disappearance of nuclear foci that mark Rad51 assemblies on damaged DNA, whereas a mutation that enhances Rad51 binding has the opposite effect. This provides a first example wherein mutations in a direct Rad51 interaction partner and mediator protein, Brca2, affect the kinetics of Rad51 focus dissolution in vivo without abrogating HR, suggesting that the events necessary for the prosecution versus the termination of HR are distinct.

Interestingly, our results imply that this mechanism affects the timing of mitotic entry. In cells wild-type for Rad51 and Brca2, nuclear envelope breakdown and entry into mitosis are accompanied by the disappearance of Rad51 foci, even when the G2 checkpoint is suppressed with caffeine, suggesting that the removal of these structures by a mechanism independent of DNA damage sensing is a prerequisite for mitotic progression. Indeed, cells carrying *Brca2* mutations that accelerate the disappearance of Rad51 foci also exhibit quicker entry into mitosis, whereas a mutation that slows the disappearance of the foci has an opposite effect. Collectively, our findings link Rad51 binding by the C terminus of Brca2, via effects on the rate of dissolution of Rad51 foci, to the timing of mitotic entry.

The cellular mechanisms that mark the end of HR during interphase remain uncertain. Moreover, it is unclear whether DNA-bound assemblies of recombination proteins must be cleared from chromatin to make way for the transactions that lead to chromosome condensation and segregation during mitosis. Our work addresses both of these issues, providing evidence for a mechanism in which the C-terminal motif in Brca2 helps time mitotic entry by controlling the dissolution of DNA-bound Rad51 assemblies. It seems plausible from in vitro biochemistry [27, 28], as well as our in vivo work on mutants, that CDK-mediated phosphorylation of the C-terminal BRCA2 motif at the end of interphase regulates its interaction with RAD51. If so, the modification may conceivably be executed in vivo by mitotic cyclin-CDK1 complexes rather than by the S phase cyclin-CDK2 complexes active during interphase. As we have previously proposed [52], the modification of BRCA2 by phosphorylation in this way may represent a mechanism integral to progression from G2 into the early stages in mitosis, marking the termination of interphase processes like HR while paving the way for new functions associated with chromosome segregation.

## Experimental Procedures

For detailed experimental procedures, please see Supplemental Experimental Procedures.

### Cell Culture and Transfection

293T cells were cultured in Dulbecco's modified Eagle's medium supplemented with 10% fetal bovine serum at 37°C. Transfection was performed with Lipofectamine 2000 (Invitrogen) according to the manufacturer's instructions. DT40 cells were maintained in RPMI 1640 medium (GIBCO-BRL) supplemented with 10^−5^ M β-mercaptoethanol, 10% fetal calf serum, and 5% chicken serum (GIBCO-BRL) at 39°C [53]. To enrich for mitotic cells, 293T and DT40 cells were treated with 120 ng/ml nocodazole (Sigma) overnight. DT40 cells were synchronized at the G1/S boundary by the addition of mimosine (Sigma) at 400 μM for 4 hr. Cells were released by washing three times with warm media, and samples were taken at indicated time points after release. Roscovitine (Sigma) was used at a final concentration of 50 μM. The doubling time of DT40 cells was determined by counting the viable cell number with a Vi-CELL Cell Viability Analyzer (Beckman Coulter) according to the manufacturer's guidelines. Growth curves were plotted with data from three independent samples.

### Plasmids and Gene Targeting

The construction of plasmids and targeting vectors used is described in Supplemental Data. Table S1 lists the sequences of the oligonucleotide primers used.

### DT40 Transfection

All targeting constructs were transfected into DT40 as described previously [53]. Briefly, 10^6^ cells were washed with 1× PBS and resuspended in a final volume of 400 μl 1× PBS. Forty micrograms of targeting construct was linearized with KpnI and dissolved in 400 μl 1× PBS. Cells and DNA were mixed in an electroporation cuvette (Bio-Rad) and kept on ice for 10 min. Electroporation was performed with a Bio-Rad Gene Pulser II at 950 μF and 250V followed by 10 min incubation on ice. Cells were seeded into five 96-well plates, and the relevant antibiotics were added 24 hr later at final concentrations of 1.5 mg/ml G418 (GIBCO) and 30 μg/ml blasticidin (InvivoGen). Clones were selected 2 weeks later. Southern blotting procedures are described in Supplemental Data.

### Generation of *Brca2* Mutants in DT40

The establishment of cell lines used in this study is described in Supplemental Data.

### Immunofluorescence and Antibodies

Immunofluorescence analyses and antibodies used in this study are described in Supplemental Data.

### Quantitative Immunofluorescence Microscopy

Images were acquired with a Zeiss LSM 510 Meta confocal microscope and quantitatively analyzed with a Cellomics HCS ArrayScan VTI (Thermo Fisher). Automated microscopy was conducted with an Olympus ScanR high-content screening microscope with a 40× nonimmersion lens. See Supplemental Data for detailed procedures.

### Sister Chromatid Exchange Assay

The sister chromatid exchange assay is described in Supplemental Data.

### Immunoglobulin Gene Diversification Assay

The wild-type, *Brca2^S3239A/S3239A^*, and *Xrcc^−/−^* cell lines were analyzed via the immunoglobulin gene diversification assay as outlined in Supplemental Data.

### Cell-Cycle Analysis and MPM2 Staining

Cells were fixed and stained with anti-MPM2 antibody and Alexa Fluor 488-conjugated secondary antibody and subjected to fluorescence-activated cell sorting (FACS) analysis as outlined in Supplemental Data. Mitotic cells were identified as those positive for MPM2 staining.

### Cell Survival Assay

Cells were plated into 96-well plates at a density of 8000 cells per well. Cell survival was measured with CellTiter-Blue reagents (Promega) following exposure to DNA-damaging agents as indicated in Supplemental Data.

### Protein Expression and Purification

Wild-type, T3232A, S3239A, and the double-mutant versions of *Gg*Brca2^(3217 aa–3252 aa)^ were transformed into *E. coli* BL21 cells (Stratagene) as a carboxy-terminal fusion to glutathione S-transferase (GST) with the pGEX4T3 vector (Pharmacia). GST-tagged proteins were expressed, purified, and incubated with glutathione Sepharose 4B beads (Amersham) as described previously [52]. Tagged proteins were eluted in 50 mM Tris (pH 8.0) supplemented with 25 mM glutathione (Sigma) and dialyzed against 40 mM Tris (pH 7.4), 100 mM NaCl, 0.1 mM EDTA, and 5% glycerol overnight at 4°C.

### In Vitro Kinase Assay

Wild-type, T3232A, S3239A, and the double-mutant versions of GST fused to *Gg*Brca2^(3217 aa–3252 aa)^ were in vitro phosphorylated in CDK1 reaction buffer supplemented with 200 μM ATP, 5 μCi of [γ-^32^P]ATP, and 20 U of recombinant CDK1 (New England Biolabs) for 30 min at 30°C. Before reaction termination with SDS sample buffer, the peptides were cleaved from the GST tag with thrombin protease (Amersham) at 25°C for 2 hr. Reaction products were resolved on a 10%–20% tricine gel (Invitrogen) according to the manufacturer's instructions. The ∼5 kDa peptides were detected by silver staining (Sigma). Dried gels were exposed to a phosphorimager screen and visualized on a Fujifilm FLA-5000 processor.

## Supplemental Data

Document S1. Supplemental Experimental Procedures, Seven Figures, and One Table
