# Supplementary material for: The Carboxyl Terminus of Brca2 Links the Disassembly of Rad51 Complexes to Mitotic Entry
Source: Curr Biol. 2009 Jul 14;19(13):1075–85. doi: 10.1016/j.cub.2009.05.057 (PMC2719694; doi:10.1016/j.cub.2009.05.057)
Supplement: Document S1. Supplemental Experimental Procedures, Seven Figures, and One Table [file mmc1.pdf]

Current Biology, Volume *19*

Supplemental Data

The Carboxyl Terminus of Brca2

Links the Disassembly

of Rad51 Complexes to Mitotic Entry

Nabieh Ayoub, Eeson Rajendra, Xinyi Su, Anand D. Jeyasekharan, Robert Mahen, and Ashok R. Venkitaraman

Supplemental Experimental Procedures

Construction of Plasmids and Targeting Vectors

The C-termini of human (*Hs*B2-9; 3189aa-3418aa) and *Gallus gallus Brca2* (*Gg*B2-9; 3152aa-3398aa) were cloned from HeLa and DT40 first strand cDNA by PCR using PF1 and PR1 and PF2 and PR2 primers, respectively. PCR products were digested with *Sal*I and *Not*I and ligated into pEF/myc/nuc (Invitrogen) digested with the same enzymes to create pEFMN-*Hs*B2-9 and pEFMN-*Gg*B2-9.

Targeting vectors for knocking in the point mutations S3239A (pLoxBsr/Neo-KI S3239A), S3239E (pLoxBsr/Neo-KI-S3239E), P3240L (pLoxBsr-KI-P3240L) and T3232A (pLoxBsr-KI-T3232A) into the endogenous DT40 *Brca2* gene were constructed as follows: A left arm (2.7kb) was amplified by PCR using the primer pair PF3 and PR3 and cloned into pUC19 vector. The mutations S3239A/E and P3240L were introduced along with a diagnostic *Sac*I site (within the intronic sequence at the left arm using mutagenesis primer TG-*Sac*I). The T3232A mutation was introduced together with a diagnostic *Bse*RI site (using mutagenesis primer AT-*Bse*RI). Constructs containing the mutant left arm were then digested with *Kpn*I and *Sal*I and ligated into pLoxBsr and pLoxNeo (kind gift from Professor Shunichi Takeda, Kyoto, Japan) digested with the same enzymes. Subsequently, the observed vectors were digested with *Not*I and *Sac*I and ligated to a right arm (2.2 kb), which was amplified by PCR using the primer pair PF4 and PR4. All mutants used in this study were created using the QuickChange Multi-Site-Directed Mutagenesis Kit (Stratagene).

Two targeting vectors for knocking in *lox*P sites at the 5’ (pLoxBsr-KI 5’B2) and 3’ (pLoxNeo-KI 3’B2) of *Brca2* were constructed to delete the entire *Brca2* gene (~40 kb). pLoxBsr-KI 5’B2 was constructed as follows: a left arm was amplified by PCR using the primer pair PF5 and PR5 , digested with *Kpn*I and *Sal*I and ligated into pLoxBsr digested with the same enzymes. This construct was digested with *Xho*I and *Not*I and ligated to the right arm prepared by PCR using PF6 and PR6 primers and digested with the same enzymes. The left arm of the pLoxNeo-KI 3’B2 vector was generated by PCR using the primer pair PF7 and PR7, digesting the PCR product with *Kpn*I and *Sal*I and ligating it into pLoxNeo digested with the same enzymes. This construct was digested with *Xba*I, blunt ended by T4 DNA polymerase and ligated to the right arm, prepared by PCR using PF8 and PR8 primers. All constructs were verified by nucleotide sequencing.

The C-terminal peptide of *Gallus gallus Brca2* (*Gg*Brca2; 3217aa-3252aa) was cloned from DT40 cDNA by PCR using PF12 and PR12 primers. PCR products were digested with *Sal*I and *Not*I and ligated into pGEX4T3, digested with the same enzymes, to create pGEX4T3-GgBrca2$^{(3217aa\text{-}3252aa)}$. Plasmids encoding the T3232A, S3239A and the combined mutants were created using the QuickChange Multi-Site-Directed Mutagenesis Kit (Stratagene).

Generation of *Brca2* Mutants in DT40

Brca2$^{S3239A/S3239A}$ cells were generated by sequential transfection of pLoxBsr-KI-S3239A and pLoxNeo-KI-S3239A targeting constructs into wild type DT40 cells. Genomic DNA was extracted from the candidate clones by DNAzol (Invitrogen) according to the manufacturer’s instructions and the identification of

successful targeting events was with *Sac*I digestion of PCR products generated by primers PF9 and PR9. The resistance cassettes were then floxed out by transient expression of Cre recombinase. Briefly, cells were transfected with pPGK-Cre plasmid (kind gift from Dr. K.J. Patel) using solution from Nucleofector Kit T according to the Amaxa guidelines. Clones with restored sensitivity to Bsr and Neo were selected. Direct sequencing of RT-PCR product of the observed clone was used to confirm S3232A mutation.

To generate a *Brca2*$^{+/-}$ cell line, wild type DT40 cells were sequentially transfected with pLoxBsr-KI 5'B2 and pLoxNeo-KI 3'B2 targeting vectors. Southern blotting combined with PFGE analysis was used to confirm the targeting of *lox*P sites at both the 5' and 3' ends of the same allele of *Brca2*. For Southern blotting genomic DNA was digested with *PshA*I and probed with a ^{32}P-labeled PCR fragment generated by PF10 and PR10 primers. The floxed *Brca2* allele was deleted by expressing Cre-recombinase as described above. Southern blotting was carried out on DNA extracted from clones with regained sensitivity to Bsr and Neo. The extracted DNA was digested with either *Ecor*V or *BsrG*I and hybridized with a 5'-probe generated by PCR using primers PF11 and PR11 or with a 3'-probe generated using primers PF12 and PR12.

The *Brca2*$^{P3240L/-}$ cell line was generated by transfecting a pLoxBsr-KI P3240L targeting construct into the *Brca2*$^{+/-}$ cell line. Targeting events were validated as described for the S3239A mutant above. The *Brca2*$^{T3232A/-}$ mutant cell line was generated using pLoxBsr-KI T3232A and validated as described above except that the PCR product was digested with *BseR*I instead of *Sac*I.

Immunofluorescence and Antibodies

Immunofluorescence analyses were done as described previously [1]. Briefly, approximately 10^5 cells were spun out onto glass slides in a Shandon Cytospin-2 cytocentrifuge for 5 min at 800 rpm slides were dried at 37°C for 5 minutes and fixed in 4% paraformaldehyde for 15 min at room temperature. Subsequently, slides were then rinsed in PBS and kept at -20°C in 70% EtOH until further analysis. Cells were blocked with 3% BSA in 1XPBS, 0.01% Tween-20, 0.01%Triton and stained with primary antibodies followed by Alexa Fluor 488 or Alexa Fluor 568 secondary antibodies (Molecular Probes, Inc.) The following primary antibodies were used: anti-RAD51 polyclonal Ab (pAb) was purchased from Merck Biosciences, anti-γ-H2AX Ser139 monoclonal antibody (mAb) from Upstate Cell Signalling Solutions (NY, USA), anti-myc mAb (9E10) from Santa Cruz biotechnology, Inc. anti-cyclinB pAb (kind gift from E. A. Nigg), anti-*Gg*Brca2 pAb (kind gift from Professor Shunichi Takeda, Japan). All Western blotting or immunofluorescence reagents were used at the dilutions recommended by the manufacturers. Imaging was performed on a Zeiss LSM510 Meta confocal microscope, using a 40x objective.

Quantitative Immunofluorescence Microscopy

Cells cytospun onto glass slides were stained with rabbit polyclonal anti-Rad51, detected using an Alexa Fluor 488-conjugated anti-rabbit antibody. Slides were mounted with Vectashield medium containing DAPI. Ten-15 representative fields were acquired using a 40x objective on a Zeiss LSM 510 Meta confocal microscope for each sample; using constant zoom and imaging parameters (laser intensities and detector settings). The collected images were batch exported as channel specific TIF files, and renamed (using r-nameit) into the Cellomics data format. The images were imported into a Cellomics HCS arrayscan VTI (ThermoFisher) under the diskscan mode; and analysed using a protocol based on the 'Compartmental analysis' bio-application. Briefly, the nuclear (DAPI) stain was used to identify objects for analysis, and intra-object changes in intensity in the Rad51 channel used to define foci. Typically, 50-500 cells were analysed in each sample to determine parameters including average number of foci per cell and number of cells with more than a defined foci count. Cells containing one or more Rad51 foci were counted as positive to ensure that cells exhibiting a complete dissolution (absence) of foci were stringently enumerated. The data were exported in Excel format and plotted in Graphpad Prism v4.0.

Slides of U2OS cells stained for cyclin B were processed by automated microscopy on an Olympus ScanR High content screening microscope using a 40x non-immersion lens. Slides were auto-focused using a stepped image intensity based algorithm for the DAPI nuclear signal, and then 25 40x fields (typically containing 1000-2000 cells) for each slide were imaged. Images of DAPI and TRITC channels were obtained for each position using real time controlled channel specific illumination from a MT20 Hg-Xenon light source, with fixed exposure times between samples. The images were stored in the ScanR format for quantitative analysis. For analysis, a constant background subtraction of 200 arbitrary units (12bit) was used across all channels. The nucleus was defined as the primary object using DAPI intensity, and using the 'watershed' function to separate closely spaced cells. The mean intensity for

nuclear cyclin B staining was determined, and cells staining two standard deviations above the population mean were scored as positive.

Sister Chromatid Exchange Assay

The sister chromatid exchange assay was performed as described previously [2]. Briefly, cells were cultured for two cell cycles in the presence of 10μM BrdU and with or without 50ng/ml mitomycin C (MMC) for the second cell cycle. Cells were pulsed with 100ng/ml colcemid for three hours before harvest. Cells were hypertonically swollen in 75mM KCl for 20 minutes at room temperature before fixation in freshly prepared Carnoy's solution (methanol: acetic acid, 3:1) for 30 minutes at room temperature. Fixed cells were dropped onto slides, dried at 50^oC for 20 minutes and then incubated for 20 minutes in 10μg/ml Hoescht-33258 (Sigma), diluted in 0.05M sodium phosphate buffer, pH 6.8 at room temperature. Slides were irradiated with UV-A (365nm) for 90 minutes and then incubated in pre-warmed 2X SSC for 1 hour at 62^oC. Slides were stained with freshly prepared Leishman's stain (Sigma) (diluted 1:3 in 0.05M sodium phosphate buffer, pH 6.8) for 2 minutes. Slides were dried and mounted with Eukitt Mounting Media (Electron Microscopy Sciences) under a coverslip. Cells in metaphase were visualised with a 100X objective on a Zeiss Axioskop 2 microscope and 50 spreads scored blind to the observer.

Immunoglobulin Gene Diversification Assay

The immunoglobulin gene diversification assay was performed as described previously [3]. Briefly, cells were stained with FITC-conjugated goat anti-chicken sIgM (Bethyl Laboratories) for flow cytometric analysis. Single cell sorting of $sIgM^+$ subclones was carried out with a Mo-Flo cell sorter (DakoCytomation) and single cells were plated to 96-well plates. 48 subclones per cell line were grown in 24-well plates and split daily. After a four week expansion, subclones were re-stained for sIgM to assess the frequency of generation of sIgM-loss variants. $sIgM^-$ cells from 12 subclones were collected using a Mo-Flo sorter and subjected to genomic DNA extraction. The VL1 sequence was then amplified by 35 cycles of PCR with CVLF5 and CVLR3 primers and cloned into pBluescript KS+ by *SacI* (in CVLF5) and *HindIII* (in CVLR3) restriction sites and transformed into subcloning efficiency DH5α competent cells (Invitrogen). Sequencing by M13F and M13R was carried out from bacterial stab cultures by Lark Technologies and MRC Geneservice and Monte-Carlo sequence analysis was performed with GAP v4.10 (Staden Package) and the MUTHR program based on an algorithm to check novel sequences against the pseudo V-genes in DT40.

Cell-Cycle Analysis and MPM2 Staining

FACS analysis was performed as previously described [4]. For MPM2 analysis, DT40 cells were washed once in PBS, fixed in cold 70% ethanol for 2 hrs, washed with PBS and stained with anti-MPM2 antibody for 1 hr at 37°C (1:1200; Upstate Cell Signalling Solutions NY, USA). Cells were then washed with 1XPBS before staining with Alexa Fluor 488 conjugated secondary antibody (1:500) for 1 hr at room temperature. Cells were stained with propidium iodide before FACS analysis. Mitotic cells were identified as those positive for MPM2 staining.

Cell Survival Assay

Cells were plated into 96-well plates at a density of 8000 cells per well. Different doses of mitomycin C (Sigma), and camptothecin (Sigma) were added, and the plates were incubated at 39°C for five doubling times. In the case of ionizing radiation, cells were exposed to different doses of IR before initial seeding. CellTiter-Blue reagents (Promega) were added to each well of the 96-well plate according to the manufacturer's guidelines. The plates were incubated at 37°C for approximately 1-2 hour in a humidified 5% CO_2 atmosphere. Number of viable cells was determined using the Fusion plate reader at A590 nm. Each experiment was done in quadruplet.

Pulsed-Field Gel Electrophoresis (PFGE) and Southern Blotting

PFGE was carried out using CHEF DR-II PFGE apparatus (BioRad) under the following conditions: 1% agarose gel in 1X TAE buffer, 200 volts at 14°C with an initial switching time of 1 s to a final switching time of 6 s for 48 hrs. For Southern blot, digested DNA samples were separated by electrophoresis and blotted on Hybond-N^+ membrane (Amersham-Pharmacia) and hybridised with probe labeled with 32-P dCTP (Amersham) by a random priming reaction [5]. After overnight hybridisation and subsequent washing, the radioactive filter was exposed to a Kodak XAR film at -80°C.

Western Blots and Immunoprecipitation

Western blot and immunoprecipitation were performed as described previously [6]. Whole-cell extracts were made in NP-40 lysis buffer. *myc*-tagged B2-9 was expressed in 293T cells and immunoprecipitated with an anti-myc monoclonal antibody. Immunoprecipitates were washed extensively in NP-40 lysis buffer and resolved on 4-12% gradient Bis-Tris gels (Invitrogen) followed by Western blotting.

Protein Expression and Purification

Wild type, T3232A, S3239A and the combined mutant version of *Gg*Brca2$^{(3217aa-3252aa)}$ peptides were transformed into *E. coli* BL21 cells (Stratagene) as a carboxyl-terminal fusion to glutathione S-transferase (GST) using the pGEX4T3 vector (Pharmacia). GST-tagged proteins were expressed, purified and incubated with glutathione-Sepharose 4B beads (Amersham) as previously described [6]. Tagged protein was eluted in 50mM Tris, pH8.0 supplemented with 25mM glutathione (Sigma) and dialysed against 40mM Tris pH7.4, 100mM NaCl, 0.1mMEDTA and 5% glycerol, overnight at 4° C.

In Vitro Kinase Assay

Wild-type, T3232A, S3239A or the double-mutant versions of GST fused to *Gg*Brca2$^{(3217aa-3252aa)}$ were *in vitro* phosphorylated in CDK1 reaction buffer, supplemented with 200 μM ATP, 5μCi of [γ-^{32}P]ATP and 20 U of recombinant CDK1 (New England Biolabs) for 30 min at 30° C. Before reaction termination with SDS sample buffer, the peptides were cleaved from the GST tag using thrombin protease (Amersham) at 25°C for 2 hr. Reaction products were resolved on a 10-20% Tricine gel (Invitrogen) according to the manufacturer's instructions. The ~5 KDa peptides were detected by silver staining (Sigma). Dried gels were exposed to a phosphorimager screen and visualized on a FujiFilm FLA-5000 processor.

Supplemental References

1. Hatanaka, A., Yamazoe, M., Sale, J.E., Takata, M., Yamamoto, K., Kitao, H., Sonoda, E., Kikuchi, K., Yonetani, Y., and Takeda, S. (2005). Similar effects of Brca2 truncation and Rad51 paralog deficiency on immunoglobulin V gene diversification in DT40 cells support an early role for Rad51 paralogs in homologous recombination. Mol Cell Biol *25*, 1124-1134.
2. Niedzwiedz, W., Mosedale, G., Johnson, M., Ong, C.Y., Pace, P., and Patel, K.J. (2004). The Fanconi anaemia gene FANCC promotes homologous recombination and error-prone DNA repair. Mol Cell *15*, 607-620.
3. Sale, J.E., Calandrini, D.M., Takata, M., Takeda, S., and Neuberger, M.S. (2001). Ablation of XRCC2/3 transforms immunoglobulin V gene conversion into somatic hypermutation. Nature *412*, 921-926.
4. Vogel, C., Kienitz, A., Hofmann, I., Muller, R., and Bastians, H. (2004). Crosstalk of the mitotic spindle assembly checkpoint with p53 to prevent polyploidy. Oncogene *23*, 6845-6853.
5. Lomonosov, M., Anand, S., Sangrithi, M., Davies, R., and Venkitaraman, A.R. (2003). Stabilization of stalled DNA replication forks by the BRCA2 breast cancer susceptibility protein. Genes Dev *17*, 3017-3022.
6. Lee, M., Daniels, M.J., and Venkitaraman, A.R. (2004). Phosphorylation of BRCA2 by the Polo-like kinase Plk1 is regulated by DNA damage and mitotic progression. Oncogene *23*, 865-872.

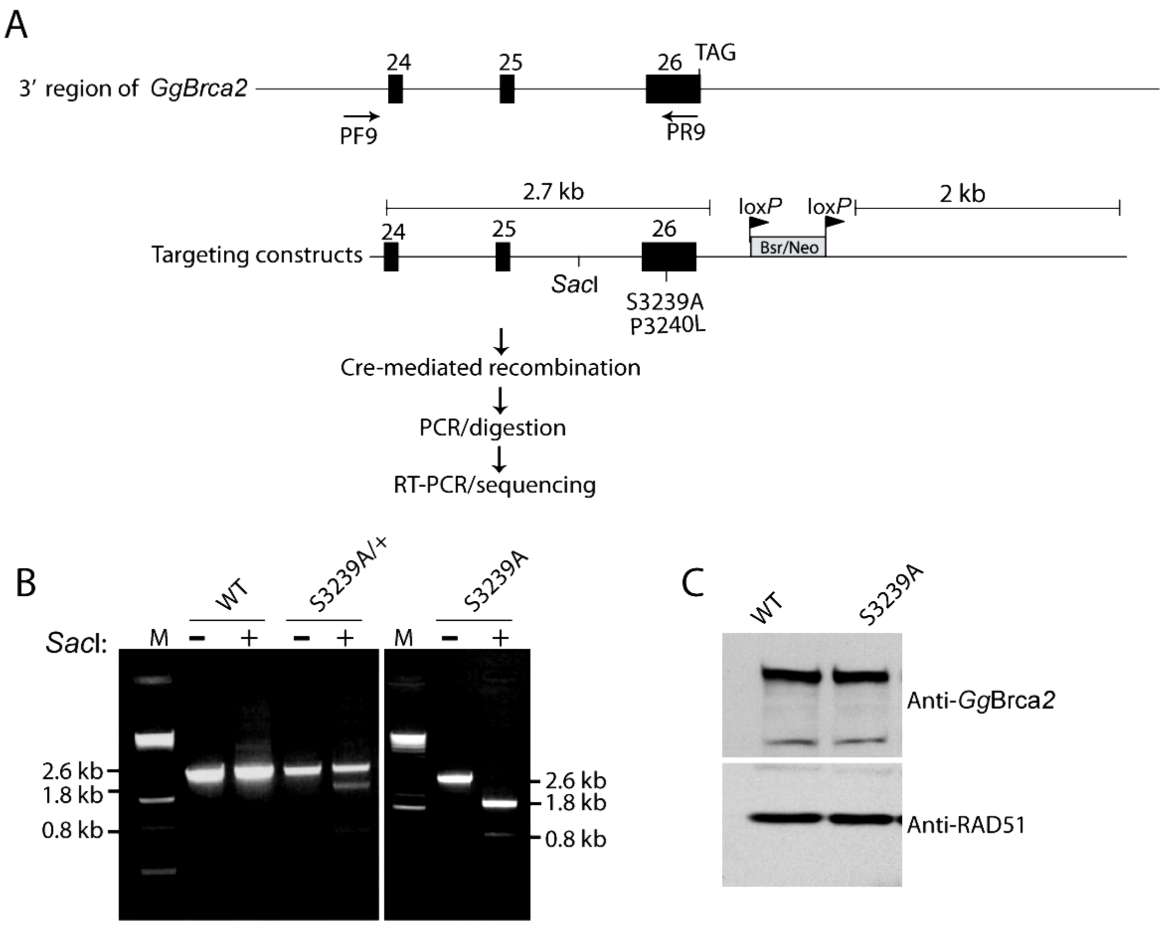

Figure S1. Hit-and-Run Gene Targeting to create the *Brca2*$^{S3239A/S3239A}$ Mutant Cell Line

(A) Schematic diagram of the targeting strategy of S3239A mutation (for details, see text and Experimental Procedures).

(B) Confirmation of S3239A knock-in by genomic PCR (using PF9 and PR9 primers) and enzymatic digestion. Successful targeting of the first allele is indicated by the presence of 1.8kb and 0.8kb bands after *Sac*I digestion in addition to the 2.6kb band from the untargeted wild-type allele. Homozygous knock-in is confirmed by complete loss of the 2.6kb band. M indicates 1Kb DNA ladder.

(C) Western blot for *Gg*Brca2 in WT and S3239A mutant cell lines shows comparable protein levels.

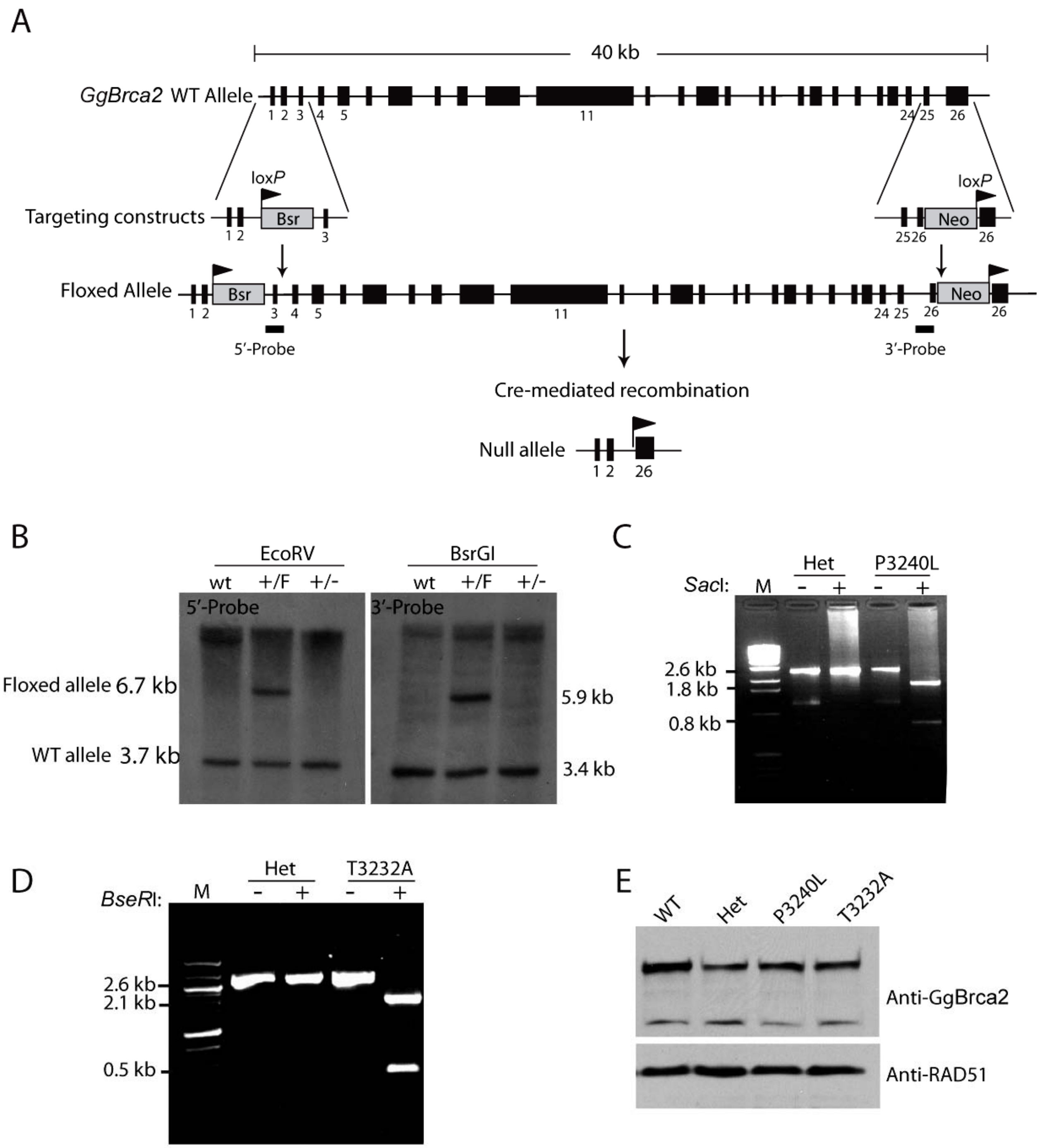
A
40 kb
GgBrca2 WT Allele
Targeting constructs
loxP
Bsr
Neo
Floxed Allele
5'-Probe
3'-Probe
Cre-mediated recombination
Null allele
B
EcoRV
BsrGI
wt +/F +/-
Floxed allele 6.7 kb
WT allele 3.7 kb
5.9 kb
3.4 kb
C
SacI:
M
Het
P3240L
2.6 kb
1.8 kb
0.8 kb
D
BseRI:
T3232A
2.1 kb
0.5 kb
E
WT
Het
P3240L
T3232A
Anti-GgBrca2
Anti-RAD51

Figure S2.

Figure S2. Generation of the *Brca2*$^{+/-}$, *Brca2*$^{P3240L/-}$, and *Brca2*$^{T3232A/-}$ DT40 Cell Lines by Hit-and-Run Gene Targeting

(A) Schematic diagram of targeting strategy for knockout of one *Brca2* allele (for details see text and Experimental Procedures).

(B) Southern blot confirming successful deletion of one allele of *Brca2* to generate a heterozygous *Brca2* cell line. Presence of the targeting cassettes on the 5' and the 3' end of the *GgBrca2* gene is indicated by a 6.7kb and 5.9kb fragment respectively, in addition to the 3.7kb fragment reflecting the presence of the wild-type allele (compare the first two lanes of each blot). Southern blotting confirms the removal of the floxed region ('F' indicates 'floxed') after *Cre*-mediated recombination, as indicated by the disappearance of the 6.7kb and 5.9kb products compared to the unfloxed heterozygous cell line (the last lane of each blot). The 3'-probe and 5'-probe denote radiolabelled DNA fragments used for Southern blotting (for details, see Experimental Procedures and Figure S2A).

(C) Confirmation of P3240L knock-in by genomic PCR and enzymatic digestion. Successful targeting of the P3240L substitution is indicated by the presence of a 1.8kb and 0.8kb bands after *Sac*I digestion compared to the intact 2.6kb band from the untargeted wild-type allele. M indicates 1Kb DNA ladder.

(D) Confirmation of T3232A/- knock-in by genomic PCR and enzymatic digestion. Successful targeting is indicated by the presence of a 2.1kb and 0.5kb bands after *BseR*I digestion versus a 2.6kb band in the heterozygous cells.. M indicates HyperLadder I DNA ladder (Bioline).

(E) Western blot for *Gg*Brca2 in *WT*, *Brca2*$^{+/-}$, *Brca2*$^{P3240L/-}$ and *Brca2*$^{T3232A/-}$ cell lines reveal comparable levels of Brca2 protein in the heterozygote backgrounds, which is approximately half the level in wild-type parental cells.

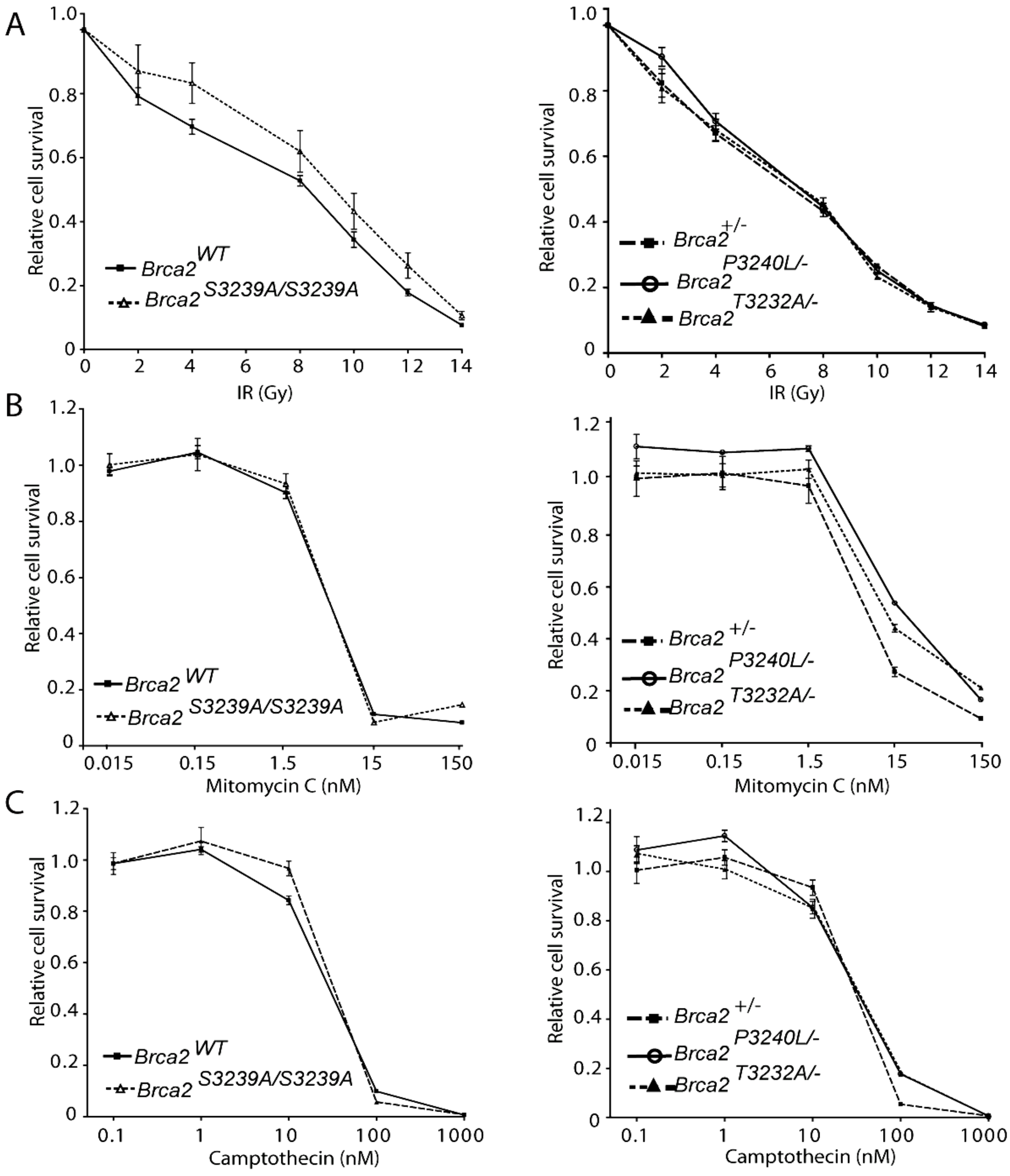

Figure S3. Cell Survival Following DNA Damage Is Intact in the *Brca2*$^{S3239A/S3239A}$, *Brca2*$^{+/-}$, *Brca2*$^{P3240L/-}$, and *Brca2*$^{T3232A/-}$ Cell Lines

Cell viability, as determined by a CellTitre Blue colorimentric assay, is expressed as a fraction of an untreated control population at each time point (see Experimental Procedures for details). Relative cell survival is plotted as a function of the indicated doses of IR (A), mitomycin C (B) or camptothecin (C). Each data point represents the mean (± SEM) of quadruplicate observations.

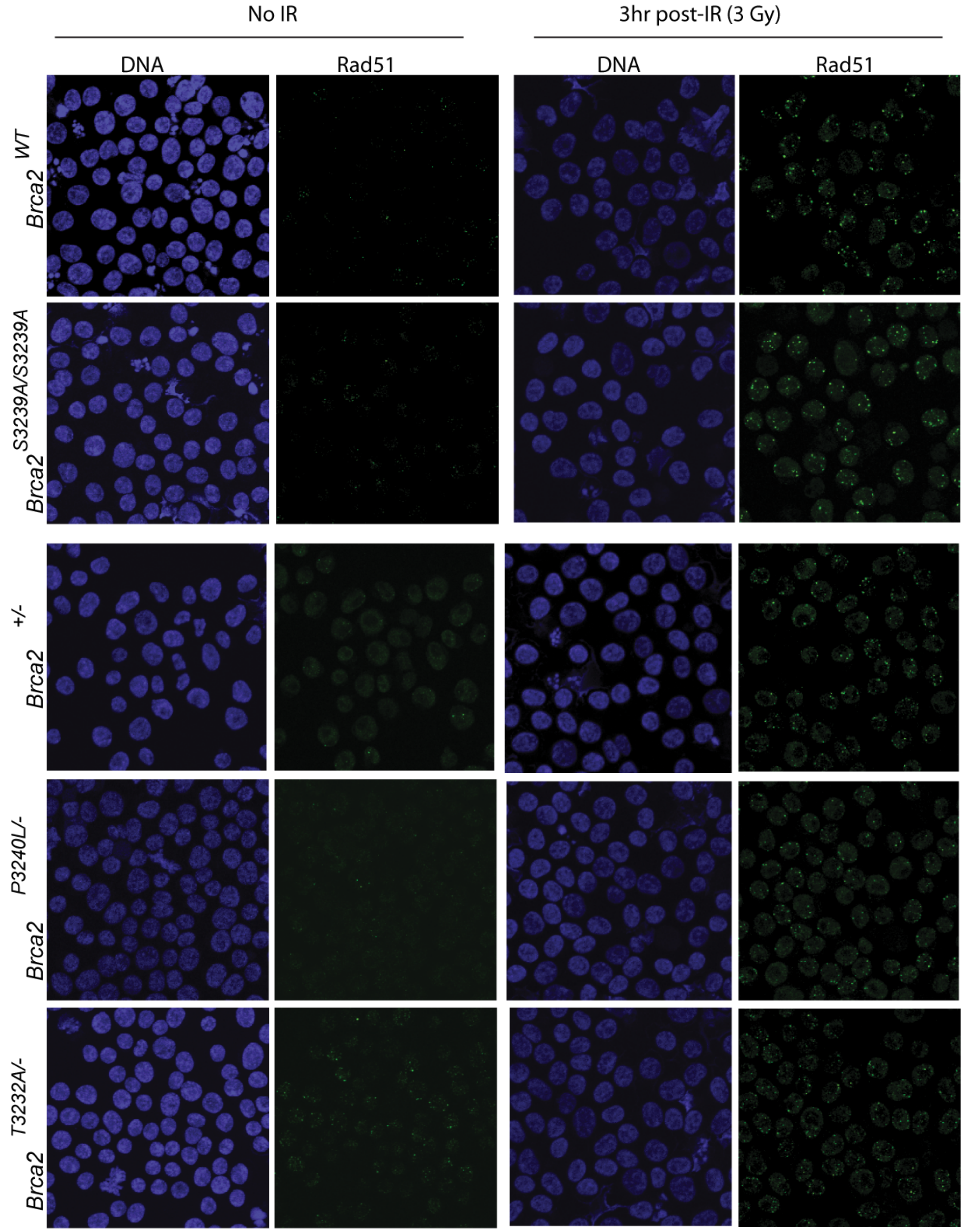

Figure S4. Normal Induction of Damage-Induced Rad51 Foci in WT, *Brca2$^{S3239A/S3239A}$*, *Brca2$^{+/-}$*, *Brca2$^{P3240L/-}$*, and *Brca2$^{T3232A/-}$* Cell Lines

Immunofluorescence staining with anti-Rad51 (green) in WT, *Brca2$^{S3239A/S3239A}$*, *Brca2$^{+/-}$*, *Brca2$^{P3240L/-}$* and *Brca2$^{T3232A/-}$* cell lines, both before, and 3hr after, 3 Gy ionising radiation (IR). DNA is stained with DAPI (blue). Rad51 foci are induced to a similar level in all cell lines.

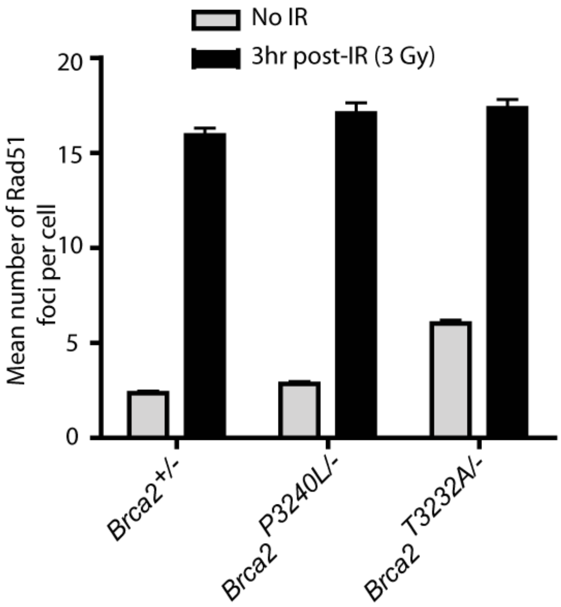

Figure S5. Intact Rad51 Foci Induction in Response to DNA Damage in *Brca2*$^{+/-}$, *Brca2*$^{P3240L/-}$, and *Brca2*$^{T3232A/-}$ Cells

Histogram showing the mean (+SEM) number of Rad51 foci per cell in the indicated cell lines (n=500 cells for each; estimated by Arrayscan analysis) before and 3 hours after IR exposure.

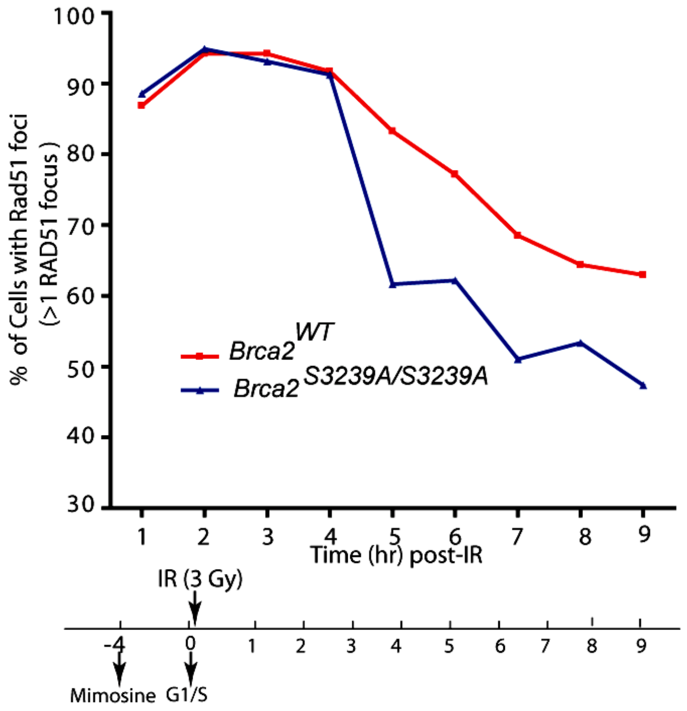

Figure S6. Faster Dissolution of Damage-Induced Rad51 Foci in *Brca2*$^{S3239A/S3239A}$ Cells Compared to Wild-Type Cells

Kinetics of Rad51 focus formation and dissolution in mimosine-synchronized WT or *Brca2*$^{S3239A/S3239A}$ cells after induction by exposure to 3 Gy IR. 500 cells were analysed on the Cellomics Arrayscan for each data point and the percentage of cells positive for Rad51 foci plotted as a function of time (see Experimental Procedures for details). Nearly 100% of cells form Rad51 foci 3 hrs after damage. However, decreased binding to Rad51 in the *Brca2*$^{S3239A/S3239A}$ cell line correlates with a faster reduction in foci-positive cells compared to the WT control.

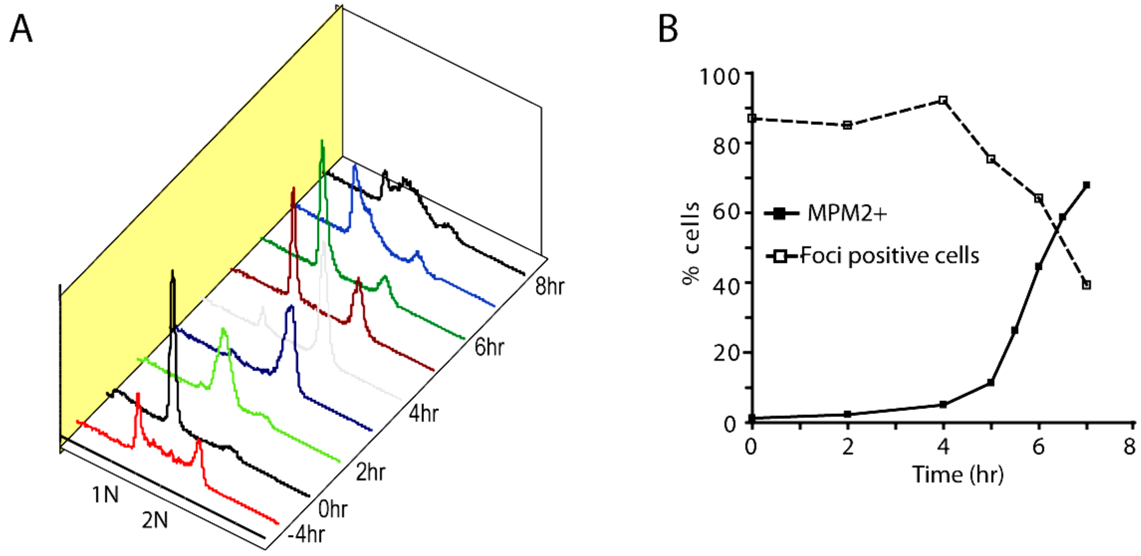

Figure S7. The Percentage of Rad51 Foci-Positive Cells Correlates Inversely with Mitotic Entry

(A) Cell cycle profiles of WT DT40 cells synchronized using mimosine at the G1/S boundary before release into the S phase. DNA content measured by propidium iodide staining and flow cytometry is plotted on the horizontal axis against relative cell number. 2*N* and 4*N* peaks represent the G1 and G2/M phases respectively.

(B) Samples collected at the indicated times were co-stained with anti-Rad51 and anti-MPM2. The cumulative percentage of cells positive for Rad51 foci or MPM2 staining (signifying mitotic entry) is plotted on the vertical axis against time after release into the S phase.

Table S1. Oligonucleotide Primers Used for PCR

Primer Name	Sequence 5'→3'
PF1	AGGTCGACAAGTGGTCCACCCCAACTAAAGACTG
PF2	ACGCGTCGACGAATGCTCTTCTCCTTCCTGCAACTCA
PF3	GCGTGAATGCGGCCGCTAGATGCACATGTACAGTACTGTGTA
PF4	CGCGGTACCTTCAGTGGCAGTCTGAATTCAGATCAGA
PF5	ATAGGTACCGTGTCAGCCGGTCACCTCCTTCTGG
PF6	ATACTCGAGGCATGCTGCTGGCTCATGGCCAACC
PF7	CGCGGTACCATCGAAGATATCGTTGTCCGCTGTT
PF8	GCTCTAGATGTGCTGTCTTAGTGTGCATGT
PF9	GTGATCCTGAGTATCTGGCGTCCAC
PF10	CAATAGTTGCACCTGCACAGCCTTCT
PF11	GTGATCCTGAGTATCTGGCGTCCAC
PF12	ACGCGTCGACAAAGCAGTGGACTTCCTCAGCTGCATAC
PR1	GGCCGCGGCCGCATATTTTTTAGTTGTAATTGTGTCCTGC
PR2	ATAAGAATGCGGCCGCTTTGCGGGATTTGCGCCTTTGCAGC
PR3	CGAGCTCGCAATGGCCTTAACATAGCAGGAAGTC
PR4	ACGCGTCGACCTAGTAGCATTTGCGGGATTTGCGCCTTTG
PR5	TATCGTCGACCTGTGGCCAAGGCCAATGGTATCC
PR6	ATCAGCATGCGGCCGCAGTTGATGCCAACTGGCTGTCCGTAG
PR7	ACGCGTCGACACATGCACACTAAGACAGCACATT
PR8	CTGAGCTCGGTACAGAACTGTGCCAGAATTACAGAGG
PR9	GCCACAAGATTATAGCTTTCATCAG
PR10	TCAGCGCCGAACTCACGAACTGAGC
PR11	GCCACAAGATTATAGCTTTCATCAG
PR12	ATAAGAATGCGGCCGCCAAACGTCGCGGAGGCTGAAATGC
TG-SacI	CCTCTCCATTTAGCTTTGAGCTCAGACACAGCGTAAATG
AT-BseRI	CATACCTGCCCCTCCTCCACTGGCACCGCTCTGTTCCATCATTT
CVLF5	CAGGAGCTCGGCTCTGTCCCATTGCTGCGCGG
CVLR3	GCGCAAGCTTCCCCAGCCTGCCGCCAAGTCCAAG
